# Uncertainty modulated exploration in the trade-off between sensing and acting

**DOI:** 10.1371/journal.pone.0199544

**Published:** 2018-07-06

**Authors:** Sonal Sengupta, W. Pieter Medendorp, Peter Praamstra, Luc P. J. Selen

**Affiliations:** 1 Donders Institute for Brain, Cognition, and Behaviour, Radboud University Nijmegen, Nijmegen, The Netherlands; 2 Department of Neurology, Radboud University Nijmegen Medical Centre, Nijmegen, The Netherlands; University of Exeter, UNITED KINGDOM

## Abstract

Many sensorimotor activities have a time constraint for successful completion. In this case, any time devoted to sensory processing is at the expense of time available for motor execution. Earlier studies have explored how this competition between sensory processing and motor execution is resolved by using experimental designs that segregate the sensing and acting phase of the task. It was found that participants switch from the sensing to the acting stage such that the overall (sensorimotor) uncertainty in the outcome of the task is minimized. An unexplained observation in these studies is the substantial variability in switching times. We investigated the variability in switching time by correlating it with the underlying sensorimotor uncertainty. To this end, we used a modified version of the virtual ball catching paradigm proposed by Faisal & Wolpert (2009). Subjects were instructed to catch a ball, but as soon as they initiated their movement the ball disappeared. We extended the range of horizontal velocities and used two different start positions of the ball to quantify the dependence of sensory uncertainty on ball velocity. Velocity dependence of sensory uncertainty allowed us to manipulate sensory uncertainty and hence the sensorimotor uncertainty. We found that the variability in switching times is correlated with two factors. Firstly, variability in switching times is greater when variation in switching time has only minimal effects on sensorimotor uncertainty. Secondly, variability in switching times is greater when the sensorimotor uncertainty is larger. Our results suggest that the variability in switching time reflects an uncertainty-driven exploratory process.

## Introduction

Most of our daily activities have a time constraint for successful completion and involve asynchronous processing of noisy sensory information and the generation of actions with uncertain outcomes [1]. Consider an object falling from a shelf. To successfully catch the object, it has to be viewed for some time before a reaching movement can be initiated. This results in a competition between the time allocated to sensing and the time spent on acting, described in two earlier studies as a sensorimotor trade-off [2,3]. To examine this trade-off, both studies used a paradigm in which the total trial time was held constant, but the sensing and acting stages were segregated, such that no sensory information is available in the acting stage. Subjects therefore had to decide when to switch from sensing to acting, denoted as the switching time.

**Fig 1:**
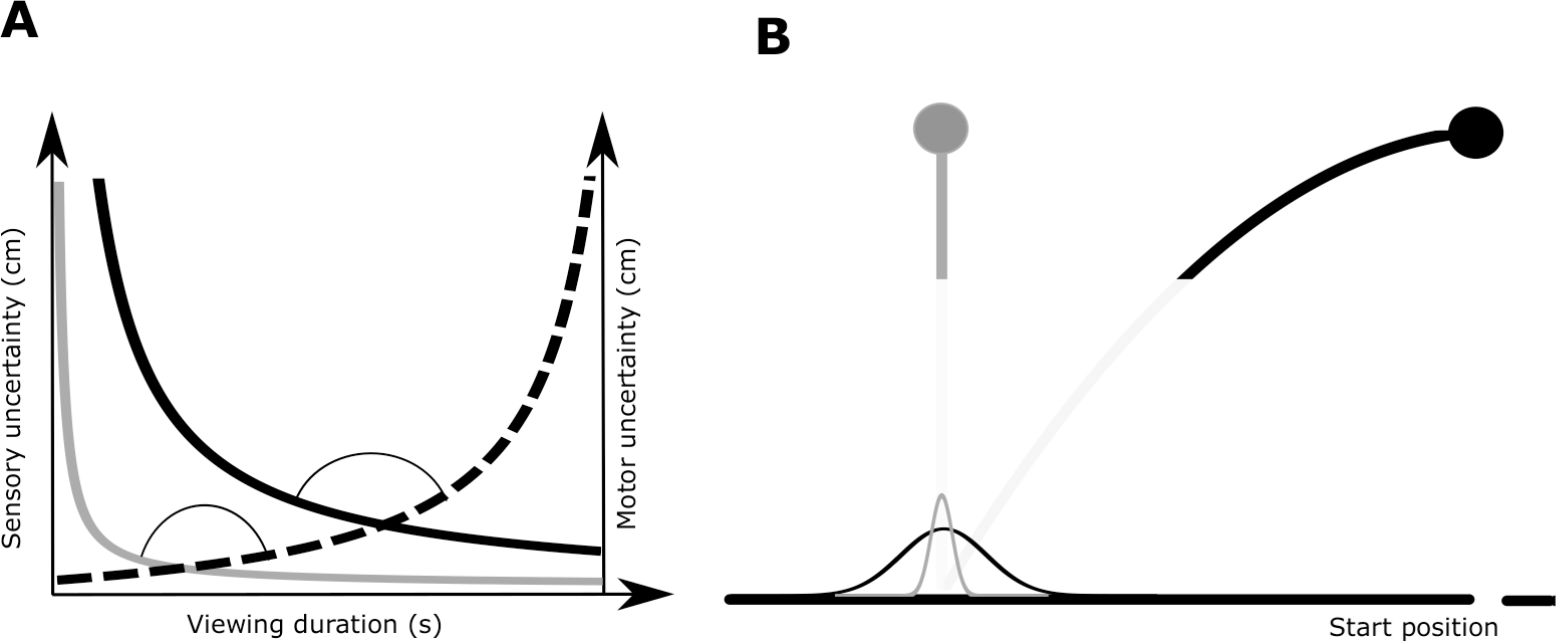
Conceptual framework of the sensorimotor tradeoff in a virtual ball catching paradigm. A. Illustrative example for two trials in which the ball’s landing position is the same. The black and gray lines depict the decrease of sensory uncertainty over time for two different rates of change of sensory information. The dashed line indicates that motor uncertainty increases with increasing viewing duration and thus less time remains for movement execution. The two trials have different times at which the sensory and motor uncertainty intersect. The uncertainty at the intersection point is the minimum attainable sensorimotor uncertainty and the angle between the sensory and motor uncertainty curves determines the shallowness of the valley and thus the sensitivity of sensorimotor uncertainty to switching time variability. B. The uncertainty in the estimate of the landing position of a ball is greater for a ball projected with greater velocity, signified by the width of the Gaussian curves. The ball falling straight down (no horizontal velocity) is linked to the gray sensory uncertainty curve in A, whereas the ball starting to the right of the landing position, and thus having a greater horizontal velocity, is linked to the black sensory uncertainty in A. The starting position of the paddle being held constant, the amplitude of the movement, and hence the motor uncertainty curve, remains the same in both cases. Hence, by changing the starting position of the ball and the horizontal ball velocity we have trials with different minimum sensorimotor uncertainty and shallowness of the valley.

Fig 1A shows how the sensory and motor uncertainty evolve as a function of time. Essentially, in each trial the sensory uncertainty decreases with time, while motor uncertainty increases with time. The combined sensorimotor uncertainty, which is the sum of the sensory and motor uncertainty, is shaped like a valley and has a minimum. An ideal performer would aim to minimize the uncertainty in the outcome of the task, hence switch such that the sensorimotor uncertainty is at its minimum. Previous studies [2,3] report that subjects, like the ideal performer, choose switching times such that the overall sensorimotor uncertainty is minimized.

However, an unexplained observation in these studies is the substantial variability in switching times, which is about twice the variability observed in reaction times for initiating normal goal directed reaching movements [4,5]. Although switching time and reaction time are not the same, both likely reflect to some extent the underlying decision processes [6,7]. Variability in switching time may thus be informative of the process of minimizing sensorimotor uncertainty.

Theoretically, switching time variability could reflect a time interval during which the combined sensorimotor uncertainty plateaus around its minimum. As illustrated in Fig 1A, the valley is formed by the combination of sensory uncertainty, which decreases with time and motor variability, which increases with time (because time available for movement decreases). If the sensory and motor uncertainty curves decrease sharply and plateau, we have a wide minimum valley. As a result, large fluctuations in switching time result in minor modulation in the uncertainty of the task outcome. In other words, the shallowness of the valley is a measure of the sensitivity of the uncertainty in the task outcome to the variability in switching time. Both Battaglia and Schrater [2] and Faisal and Wolpert [3] suggest that the variability in switching time is a consequence of the shallowness of the valley.

In addition to the absence of changes in performance due to changes in switching time, the level of the overall uncertainty in outcome may also affect switching time variability [8,9]. Studies using reinforcement learning paradigms have shown that larger uncertainty in task outcome drives stronger exploratory behavior [10,11]. Applying this principle to sensorimotor trade-off tasks, the range of switching times may increase if the minimum sensorimotor uncertainty becomes larger.

In this study, we investigate how valley shallowness and uncertainty about the task outcome affect the variability in switching time. To this end we need a paradigm where the minimum sensorimotor uncertainty (or uncertainty in the task outcome) and the shallowness of the valley can be manipulated, without introducing artificial noise to the sensory and/or motor system (e.g.[12]) as illustrated in Fig 1A. Because, for a given movement amplitude, the motor uncertainty curve as a function of time is fixed, the uncertainty about the task outcome and the shallowness of the valley can only be manipulated by the sensory uncertainty curve (illustrated in Fig 1A). Therefore, we modified the virtual ball catching paradigm used by Faisal & Wolpert [3] to address our question.

In the virtual ball catching paradigm, a ball is projected under simulated gravity with an initial horizontal velocity. Subjects are instructed to catch the ball at the ground level using a paddle. However, the ball disappears as soon as the subjects start the movement. While the original paradigm did not factor in the role of ball velocity on sensory uncertainty, we used two ball start positions and a wider range of horizontal velocity to manipulate sensory uncertainty.

In support of such velocity dependence, previous studies suggest that uncertainty of object velocity increases with its velocity [13], and that discriminability of object acceleration deteriorates with its speed [14]. Also, the interception literature reports that speed of the target object influences the kinematics of the catching movements [15,16]. Accordingly, Fig 1B illustrates that for the same viewing duration, greater horizontal ball velocity leads to increased uncertainty in the estimate of the landing position. The dependence of sensory uncertainty on ball velocity provides an important design advantage: Two balls with the same landing position, but with different horizontal velocities (see Fig 1B) result in different sensory uncertainty functions (Fig 1A). Hence, the dependence of sensory uncertainty on horizontal ball velocity provides a means to manipulate the uncertainty about the task outcome and the shallowness of the valley (for detailed explanation see the methods section *‘Motivation for modifications of Faisal & Wolpert’s paradigm’*).

Consistent with previous studies [2,3], we observed large variability in chosen switching times. This variability was typically greater for the trials in which the ball was projected with greater velocity, irrespective of the amplitude of movement required to catch the ball. As predicted, we found that the sensory uncertainty of the task outcome, was modulated by ball velocity. In the combined task, the velocity dependence of sensory uncertainty affected the switching time variability through its effects on both the rate of change of sensorimotor uncertainty (the shallowness of the valley) and the minimum sensorimotor uncertainty on each trial. The consistent patterns observed in the variability in switching time across subjects suggest the involvement of an active, uncertainty mediated exploration process.

## Methods

### Subjects

Ten naïve right-handed subjects (6 male, 18–26 years) with normal or corrected to normal vision performed the experiment. Prior to the experiment, subjects provided written informed consent after receiving detailed written and verbal information. Experiments were approved by the local ethics committee of the Faculty of Social Sciences, Radboud University Nijmegen, The Netherlands. Subjects were given monetary compensation (10 € / hour) or course credits for their time (2 sessions of 2.5 hours).

### Setup

Subjects were seated in front of a planar robotic manipulandum (vBOT [17]). The visual scene was drawn on a screen (model VG278H, Asus, Taiwan) which was updated at 120 Hz. A semi-silvered mirror was used to project the visual scene into the action plane such that the subjects could look into the visual scene. Subjects used their right hand to make reaching movements, while leaning forward and looking down at the visual scene with their forehead leaning on a headrest. The hand was not visible to the subject, but its position was visible at any instant as a red rectangle, representing a paddle used to catch the ball. Hand movements were constrained, using a force channel [18], onto a horizontal line. Kinematic data was measured at 1000 Hz.

### Tasks

**Fig 2:**
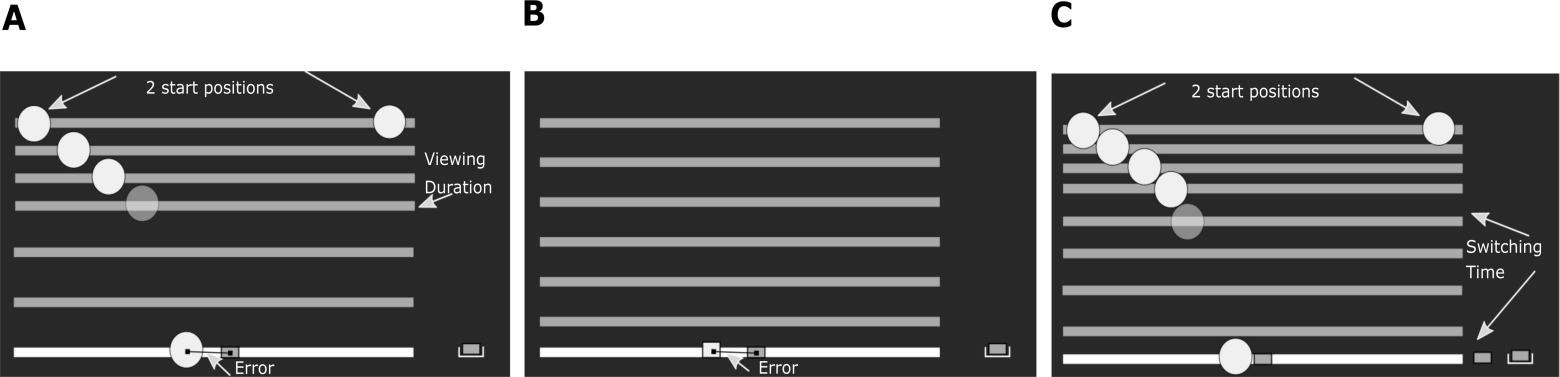
Sketch of the experimental conditions. During the experiment we used green color for the ball and red color for the rectangular paddle. A. Sensory task: The subject would see a ball falling, with a constant horizontal velocity and under simulated gravity, from either the left or right start position (fixed within a session). Together with the ball a horizontal bar fell. At some point the ball disappeared, but the bar kept falling. After the bar hit ground level (white line), the subject had infinite time to indicate the estimated landing position, using the red paddle. B. Motor task: A reach target is shown as a small red square at ground level. The required movement time is first indicated by a bar falling at constant velocity. Next, the subject has to reach from the start position to the target within the prescribed movement time, again with the bar falling as an indicator of remaining time. C. Combined task: The ball falls from either the left or right start position (fixed within a session), just like in the sensory task (A). In the combined task the ball disappears when the subject switches from the sensing to the acting stage (denoted as the switching time in the figure) by moving the paddle outside the starting position. The task of the subject is to position the paddle underneath the ball before it hits ground level. In all conditions the subject receives error feedback about the final paddle position relative to the true landing position of the ball (A, C) or reach target (B).

Each subject completed two sessions of three tasks each: a sensory task, a motor task and a combined task. Fig 2 shows a grayscale schematic of the screens presented to the subjects in the three tasks. The only difference between the two sessions was in the start position of the ball. During the sensory task and the combined task, the subject saw a simulation of a ball appearing in the top-left or top-right corner of the screen and moving in a parabolic trajectory on the screen. The ball (shown as a green filled circle with a radius of 0.5 cm) would always land on the ground, represented as a white line 30 cm long on the bottom of the screen. The ball would fall from a height of 25 cm relative to the ground, under simulated gravity (g = 25.5 cm/s^2^) with no initial velocity in the downward direction, but with a constant velocity in the horizontal direction. This resulted in a flight duration of 1.4 s. In the motor task, the reach target was indicated by a square (edge = 0.5 cm) at ground level. The start position of the paddle was at the bottom right corner of the screen and separated from the ground line by 5 cm. In the next sections, we will describe the three tasks in more detail.

#### Sensory task

The aim of the sensory task was to quantify the dependence of sensory uncertainty on horizontal ball velocity and its viewing duration (illustrated in Fig 2A). Subjects were instructed to observe a ball falling in a parabolic trajectory. The ball was projected with zero vertical velocity and a horizontal velocity randomly chosen from 0, 2, 4, 6, 8, 10, 12, 14 cm/s. The ball disappeared at the end of a predetermined viewing duration which was randomly chosen from 200, 400, 600, 750, and 950 ms. A bar, shown simultaneously with the ball, continued falling until it reached ground level at 1400 ms. To ensure that subjects estimate the end position and do not use velocity dependent strategies, their hand was clamped to the start position and was released only after the ball disappeared. They were instructed to bring the paddle (width 0.5 cm) to the estimated landing position. Once they reached the estimated position, they pressed the space bar of the keyboard with their left hand to finish the trial. This ensured that there was no time restriction on executing the movement and minimized the contribution of motor uncertainty in this sensory task.

#### Motor task

The aim of the motor task was to quantify motor uncertainty, i.e. the standard deviation of the distribution of reach endpoints, as a function of movement time and movement amplitude (illustrated in Fig 2B). Subjects were instructed to make movements of a given amplitude to be executed in a pre-defined time. Movement amplitudes were defined by 8 target positions (range 10–30 cm, corresponding to the landing positions from the sensory experiment) and were presented in random order. The required movement time was randomly chosen from 450, 650, 800, 1000, 1200 ms. The required movement time was indicated to the subject by a horizontal bar falling from the top of the screen at constant velocity until it reached ground level. After having seen this bar falling, subjects could now start their own movement. Once the subject initiated a movement (i.e. speed > 2.5 cm/s), the bar would start falling again to guide the required movement time. Thus, the bar fell twice, once to indicate the required movement time and the second time to pace the movement. The endpoint of the movement was defined as the position of the paddle at the end of the required movement time, irrespective of the movement speed.

#### Combined task

The aim of the combined task was to measure the switching time that subjects chose to catch a ball falling along a parabolic trajectory. The ball disappeared as soon as the subject initiated movement (speed > 2.5cm/s), separating the sensing and acting stages of the task (illustrated in Fig 2C). The simultaneously displayed bar continued to fall, as a timing aid. Subjects were instructed to catch as many balls as possible. Paddle widths were randomly chosen from 0.5, 1, 2, 4 cm. Although paddle width does not change optimal behavior, it helps to keep subjects motivated. The horizontal speed of the ball was chosen from a continuous and uniform distribution over the 0–15 cm/s interval. The ball hit the ground after 1400 ms. Feedback about the outcome was only given after the ball hit the ground. Visual feedback was given at the end of each trial, showing the ball’s landing position and the position of the paddle when the ball hit the ground.

#### Procedure

Subjects performed the experiment in two sessions, conducted on separate days, with a maximum interval of 14 days. The sessions differed in initial position and the direction of the horizontal ball velocity. That is, in one session the start position of the ball was in the top left corner and its horizontal velocity was always toward the initial paddle position, while in the other session it was in the top right corner and its horizontal velocity was away from the initial paddle position. The order of the two start positions was counterbalanced between subjects. Each session began with the sensory task (440 trials), in which every combination of horizontal ball velocity and viewing duration (8 velocities x 5 viewing durations) was repeated 11 times. This was followed by the combined task, with 400 trials with horizontal ball velocities drawn from a uniform distribution between 0 and 15 cm/s. The session ended with the motor task (280 trials), in which every combination of movement amplitude and movement time (8 amplitudes x 5 movement times) was repeated 7 times. Because the motor task was the same in the two sessions, this amounted to a total of 14 repetitions per amplitude-time combination. Each of the three parts of a session began with a short practice block (~ 30 trials) to familiarize the subjects with the setup and instructions. The parameters for the practice trials were chosen arbitrarily. Subjects were given a mandatory break after every 100 trials. The duration of the breaks was determined by the subject (which never exceeded 15 minutes). An experimental session lasted for approximately 2.5 hours (60 min + 40 min + 25 min + break times).

### Analysis

Following Faisal and Wolpert [3] we modeled an ideal performer in the combined task using the sensory and motor uncertainty estimated in the sensory and motor tasks. The ideal performer maximized the task outcome (catch maximum number of balls) by initiating movements such that the combined sensorimotor uncertainty is minimized.

#### Sensory task

We parameterized sensory uncertainty, σ_S_ as a multiplication of a power of the viewing duration (t) and a power of the horizontal velocity of the ball (v), σ_S_ = at^b^v^c^. A multiplicative model captured the idea that sensory information (‘velocity’) is accumulated over time to provide an estimate of the landing position. The parameter *a* represented the baseline uncertainty in the behavior. The influence of time is quantified in *b* and the effect of velocity is quantified in the parameter *c*. For fitting the data, we subtracted the mean across trials, i.e. the bias, from the end point of each movement. The free parameters of the sensory uncertainty model were fitted using maximum likelihood estimation. We computed the log-likelihood of the error being drawn from the normal distribution with mean = 0 and standard deviation given by the model. The alternative model, where the influence of time and velocity are additive, provided a worse fit to the data.

The inclusion of horizontal velocity deviates from Faisal & Wolpert (2009). To warrant the inclusion of this extra factor, we compared it to the reduced model with c = 0. The parameters that maximized the likelihood were taken as the best fit parameters. We compared a velocity dependent and independent model using the Bayesian Information Criterion (BIC).

#### Motor task

The motor uncertainty (σ_M_) depends on movement time and movement amplitude, as quantified by Fitts’ law (Fitts, 1954). We rewrote Fitts’ law in terms of motor uncertainty as $\sigma_{M}=x{∙2}^{\left(1-\frac{t-d}{e}\right)},$where *x* denotes the movement amplitude and *t* denotes the movement time. The parameters *d* and *e* are subject-specific parameters. Parameter *e* is a measure of the time after which the motor uncertainty plateaus. The ratio *d/e* is a measure of the maximum motor uncertainty for extremely short movement times. For fitting the data, we subtracted the mean across trials, i.e. the bias, from the end point of each movement. The free parameters of the motor uncertainty model were fitted using maximum likelihood estimation. We computed the log-likelihood of the error being drawn from the normal distribution with mean at zero and a standard deviation given by the model.

#### Sensorimotor task

We computed the sensorimotor uncertainty using the intrinsic sensory (σ_S_) and motor uncertainty (σ_M_) as derived from the sensory and motor task. The total time available for the trial was T, hence time available for movement was T–t. We assumed that in the combined task the sensory and motor uncertainty are independent of each other. Therefore, the expected endpoint variance of the paddle position for a given switching time in the combined task can be modeled as the sum of the sensory and motor uncertainty, if expressed in terms of variance.

$$\sigma_{\text{combined}}^{2}\left(t\right)=\sigma_{\text{S}}^{2}\left(t\right)+\sigma_{\text{M}}^{2}\left(\text{T}-t,x\right)={\left({\text{at}}^{\text{b}}{\text{v}}^{\text{c}}\right)}^{2}+{\left(x\cdot 2^{1-\frac{\left(\text{T}-\text{t}\right)-\text{d}}{\text{e}}}\right)}^{2}$$ (1)

The time at which σ_combined_(t) is minimum for a given ball velocity and landing position is referred to as the optimal switching time (*t_optimal*). An optimal performer would initiate the movement at this time such that the overall uncertainty about task outcome is minimized and the chances of catching the ball is maximized. The observed switching times were compared using a linear regression between the landing position and the observed switching time data, separately for the two ball starting positions. We also performed this regression between the landing position and the optimal switching times. We used these fit coefficients to compare the observed switching times and the predicted switching times.

Next, we computed the observed variability in switching time by binning switching times from trials that required similar movement amplitudes (each bin was 2.1 cm amplitude in width and contained approximately 40 trials). We computed the mean and variance of the observed switching times in each bin, separately for the two ball starting positions.

We evaluated the contribution of three possible factors to switching time variability. First, we consider the influence of uncertainty about task outcome on switching time variability. The outcome uncertainty is lowest when the sensorimotor uncertainty that can be attained by an ideal performer is minimal, i.e. when switching from sensing to acting in the lowest point of the sensorimotor uncertainty function (σ_combined_(*t_optimal*), as illustrated in Fig 1A).

Second, we consider the role of the sensory component (σ_combined_(*t_optimal*), as manipulated by horizontal ball velocity) on switching time variability. On each trial, the landing position the subject aimed at is drawn from a normal distribution with the standard deviation equal to the sensory uncertainty. This results in a probability density of landing positions, each associated with its own optimal switching time (as illustrated by the Gaussian about the landing position in Fig 1B). We compute the 95% confidence interval over this, asymmetric, switching time distribution as a proxy for switching time variability.

Third, we consider the contribution of the shallowness of the valley of the sensorimotor uncertainty function to the variability in switching time. As illustrated in Fig 1A, the sides of the valley are formed by the, separately derived, sensory and motor uncertainty functions. Hence, the greater the steepness of the sensory and the motor function near the optimal switching time, the sharper the valley. When the sensory and motor uncertainty functions plateau early, there is a shallow valley; that is, small fluctuations in switching time around the optimal switching time have negligible effect on the sensorimotor uncertainty. Therefore, the shallowness of the valley is a measure of the sensitivity of uncertainty in the task outcome to small variations in switching time. We quantify the shallowness of the valley for each trial at the optimal switching time as the angle between the sensory and motor uncertainty functions for that trial, following θ=arctans-m(1+s×m), where *s* and *m* are the slopes of the sensory and motor uncertainty functions at the optimal switching time.

Finally, we consider that switching time variability is determined by a combination of the above-mentioned factors. We fit a linear regression model that factors in the combined effect of the shallowness of the valley (θ) and the minimum uncertainty (σ_combined_(*t_optimal*)) on the observed variability in switching time.

$$\sigma_{Switching\;Time}=\beta_{0}+\beta_{1}\times \theta +\beta_{2}\times \sigma_{combined}\left(t\_optimal\right)$$

All analyses were performed with MATLAB 2015b. Statistical tests were performed using the statistics toolbox in MATLAB. Unless otherwise stated the alpha value is set to 0.05.

#### Motivation for modifications of Faisal & Wolpert’s paradigm

**Fig 3:**
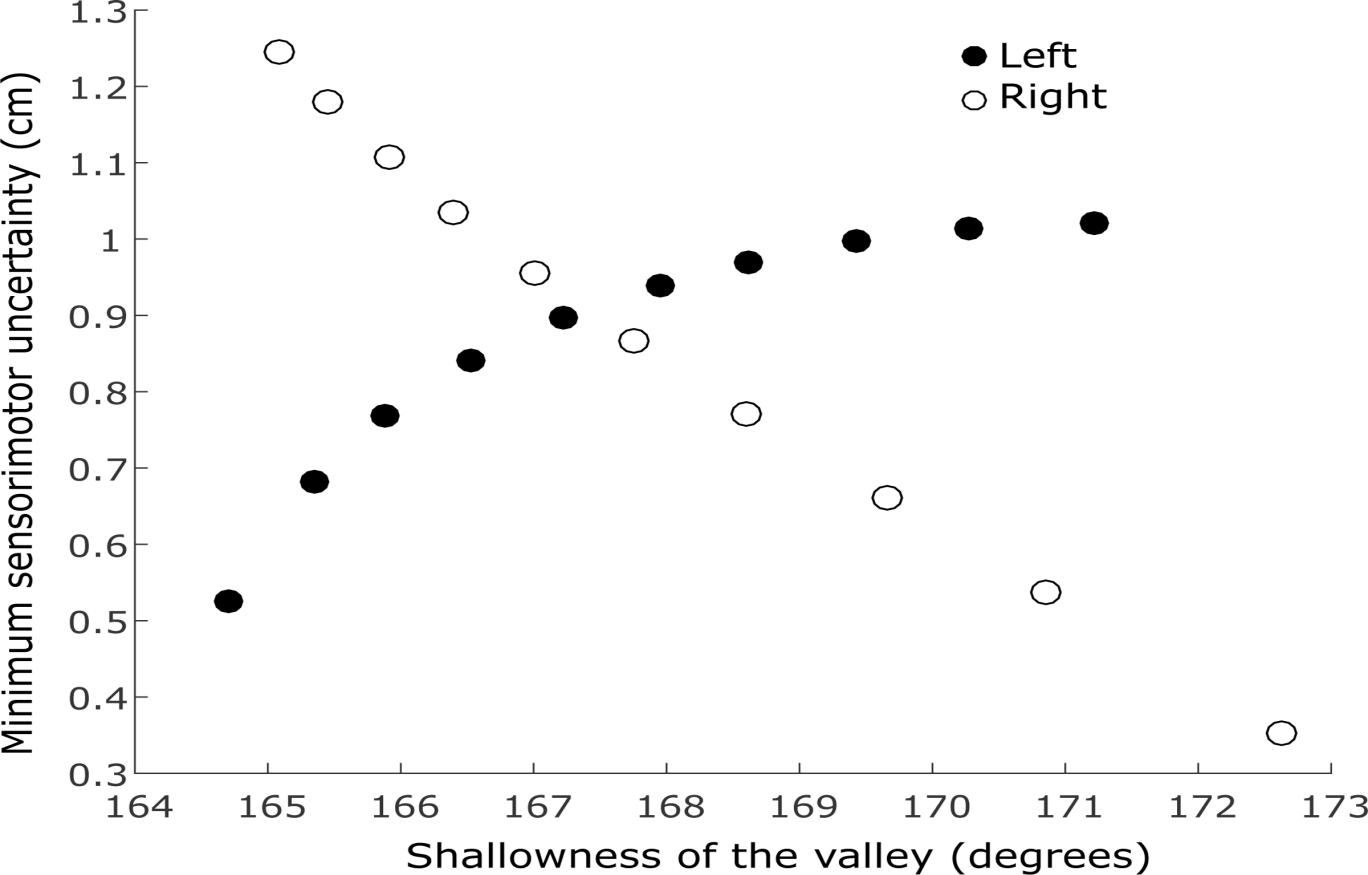
Relation between the shallowness of the valley and the minimum sensorimotor uncertainty (using an example subject data (F)). The filled dots represent the data for the session with balls starting from the left. When the ball starts on the left (filled circles), decreases in the minimum sensorimotor uncertainty are associated with increases in the shallowness of the valley (that is, the valley angle increases). In contrast, when the ball starts on the right (unfilled circles), increases in the minimum sensorimotor uncertainty are associated with decreases in the shallowness of the valley (that is, the valley angle decreases).

The paradigm and analyses described above deviate from the original description by Faisal and Wolpert [3]. We modified the original paradigm and ideal performer model for two reasons: 1. The larger range of velocities enabled us to examine a velocity dependence of the sensory uncertainty about the ball’s landing position (in the sensory task). Using two starting positions for the ball, while keeping the starting position of the subject (paddle) fixed, allowed us to verify the effect of ball velocity on sensory uncertainty. 2. In the combined task, the two starting positions, along with a possible velocity dependence, result in different optimal behaviors for balls with the same landing position but different horizontal velocities. Note that the possible determinants of the variability in switching time are computed from the sensorimotor uncertainty function. This implies that they cannot be manipulated independent of each other. Therefore, the two different starting positions of the ball are necessary to investigate the factors that may underlie variability in switching time in the combined task. Using two starting positions while keeping the start position of the paddle fixed, there are trials which require the same movement amplitude but have different sensorimotor uncertainty function because the ball has different horizontal velocity. That is, if we consider Eq 1 for trials that need the same movement amplitude for a successful catch, σ_M_ remains the same, but σ_S_ differs. σ_S_ increases with horizontal velocity of the ball, which increases with amplitude *x* when the ball starts from the right but decreases with amplitude (*x*) when the ball starts from the left. Thus, between the two start positions of the ball, there are trials which require movements of the same amplitude *x*, but have different minimum sensorimotor uncertainty and different shallowness of the valley. We illustrate this further in Fig 3: when the ball starts from the left, shallowness of the valley is a decreasing function of the minimum uncertainty. By contrast, when the ball starts from the right, shallowness of the valley is an increasing function of the minimum uncertainty. When we analyze both conditions together in a regression model, the shallowness of the valley cannot be computed as a single transformation of the minimum sensorimotor uncertainty. Therefore, comparing between the two start positions of the ball allows us to tease apart the individual effects of the possible determinants of switching time variability that we consider.

## Results

This study aimed to explain the variability observed in the switching times in sensorimotor trade-off in a ball catching task. We approached this problem in two steps. First, we model the combined sensorimotor uncertainty using individually measured sensory and motor uncertainty. From this model, we predict the optimal switching time i.e., the time at which the combined sensorimotor uncertainty is minimum. Then, we correlate the variability in switching time with the minimum uncertainty and the sensitivity of the uncertainty in the outcome to changes in switching time.

### Optimal vs. observed switching times

#### Sensory uncertainty

**Fig 4:**
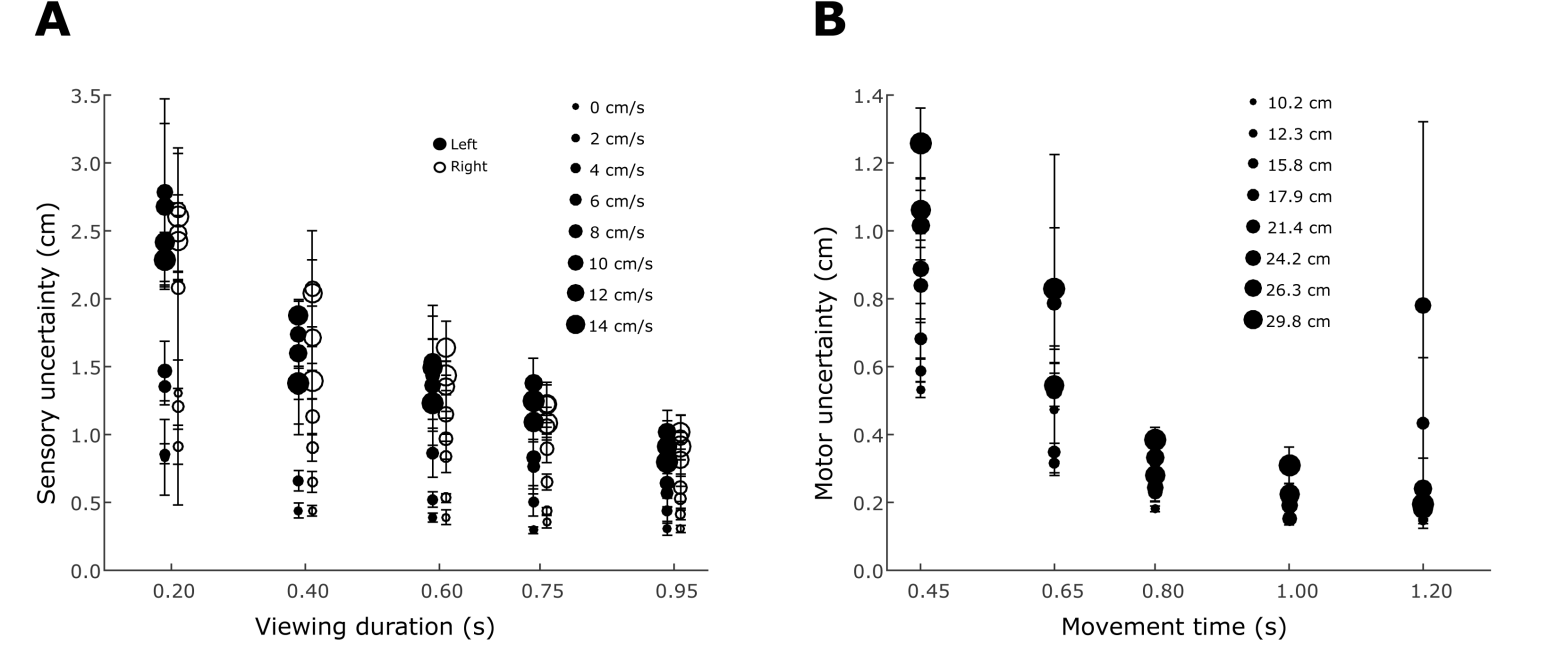
Summary of the sensory uncertainty and motor uncertainty across all landing positions and times. A. Sensory uncertainty. The horizontal velocity of the ball (which determines its landing position) is coded by the size of the marker. Empty circles represent the sensory uncertainty for the session with balls starting from the right. Filled circles represent the sensory uncertainty for the session with balls starting from the left. In both panels, the error bars represent the standard error across participants. B. Motor uncertainty. The amplitude of the movement is coded by the size of the marker.

In the sensory task, we quantified the effect of the horizontal velocity and viewing duration of the ball on the uncertainty in the estimate of the landing position of the ball. Since there was no time constraint in the sensory task, the measured uncertainty had negligible motor contribution. Fig 4A shows the influence of velocity and viewing duration on sensory uncertainty (i.e. the SD over repeated trials) for all subjects. Sensory uncertainty decreased with increased viewing duration and increased with greater ball velocity. A repeated measures ANOVA on sensory uncertainty with the factors ball speed, ball direction and viewing duration, showed main effects for both viewing duration (F (4,36) = 63.17, p<0.005) and horizontal ball speed (F (7,63) = 23.46, p<0.005) and an interaction between these factors (F (28,252) = 2.46), p<0.005). We found no significant main effect of ball direction on the sensory uncertainty (F (1,9) = 0.0002), p = .987). Hence, for all further analyses we pooled the sensory uncertainty data from the sessions with left and right-ward ball direction.

Next, we parameterized the dependence of sensory uncertainty on viewing duration and horizontal ball velocity as σ_S_ = at^b^v^c^. Table 1 shows the parameters of this fit for the individual subjects. The subject specific parameter *a* had an average value of 0.35 (SE = 0.04). The power of the viewing duration was negative in all subjects (*b* = -0.57 (SE = 0.04)) indicating that sensory uncertainty decreases with increasing viewing duration. The power of the velocity dependent term was positive in all subjects (*c* = 0.41 (SE = 0.03)), indicating that greater ball velocities result in greater uncertainty in the estimates of the landing position. A BIC analyses revealed that a parameterization of sensory uncertainty without a power law dependence on ball velocity, i.e. *c* = 0, is statistically an inferior description (average ΔBIC = 254, minimum ΔBIC = 93). The R^2^ for the sensory uncertainty model ranged from 0.42–0.93 (mean = 0.78).

**Table 1:** Model parameters, based on sensory data (a, b, c) and motor data (d, e).

	Sensory model			Motor model	
Participant	a	b	c	d	e
A	0.27	-0.70	0.38	-2.27	0.47
B	0.26	-0.74	0.26	-1.01	0.26
C	0.25	-0.56	0.47	-0.73	0.23
D	0.34	-0.67	0.42	-1.05	0.29
E	0.30	-0.78	0.35	-1.56	0.34
F	0.30	-0.51	0.54	-1.49	0.35
G	0.48	-0.28	0.45	-2.43	0.51
H	0.20	-0.62	0.48	-1.11	0.27
I	0.50	-0.43	0.57	-1.06	0.27
J	0.59	-0.45	0.24	-1.68	0.36

#### Motor uncertainty

In the motor task, we measured how motor uncertainty, i.e. the variability in motor outcome over repeated movements, depends on movement duration and movement amplitude. Fig 4B shows motor variability (i.e. the SD over repeated movements) for all subjects. Motor variability increases with movement amplitude and decreases for longer movement times. To parameterize the effects of movement amplitude and movement time on motor variability, we fit $\sigma_{M}=x{∙2}^{\left(1-\frac{t-d}{e}\right)}$to the endpoints of the movements. This model captured the motor variability in our subjects, with average R^2^ value of 0.71 (ranging from 0.11–0.96). As shown in Table 1, the parameters of the fit were consistent across subjects, with parameter *d* = -1.44 s (SE = 0.17s) and e = 0.33s (SE = 0.02s).

#### Sensorimotor uncertainty

The aim of the combined task was to observe when the subjects switched from sensing to acting. On average, subjects switched from sensing to acting after 640 ms and caught 77% of the balls when the ball started from the left (range 68%– 89%), overestimating the ball landing position by 0.16 cm (SE = 0.04 cm). On average, subjects caught 74% of the balls when the ball started from the right (range 44%– 87%). Across trials, subjects underestimated the landing position of the ball by 0.36 cm (SE = 0.13 cm).

**Fig 5:**
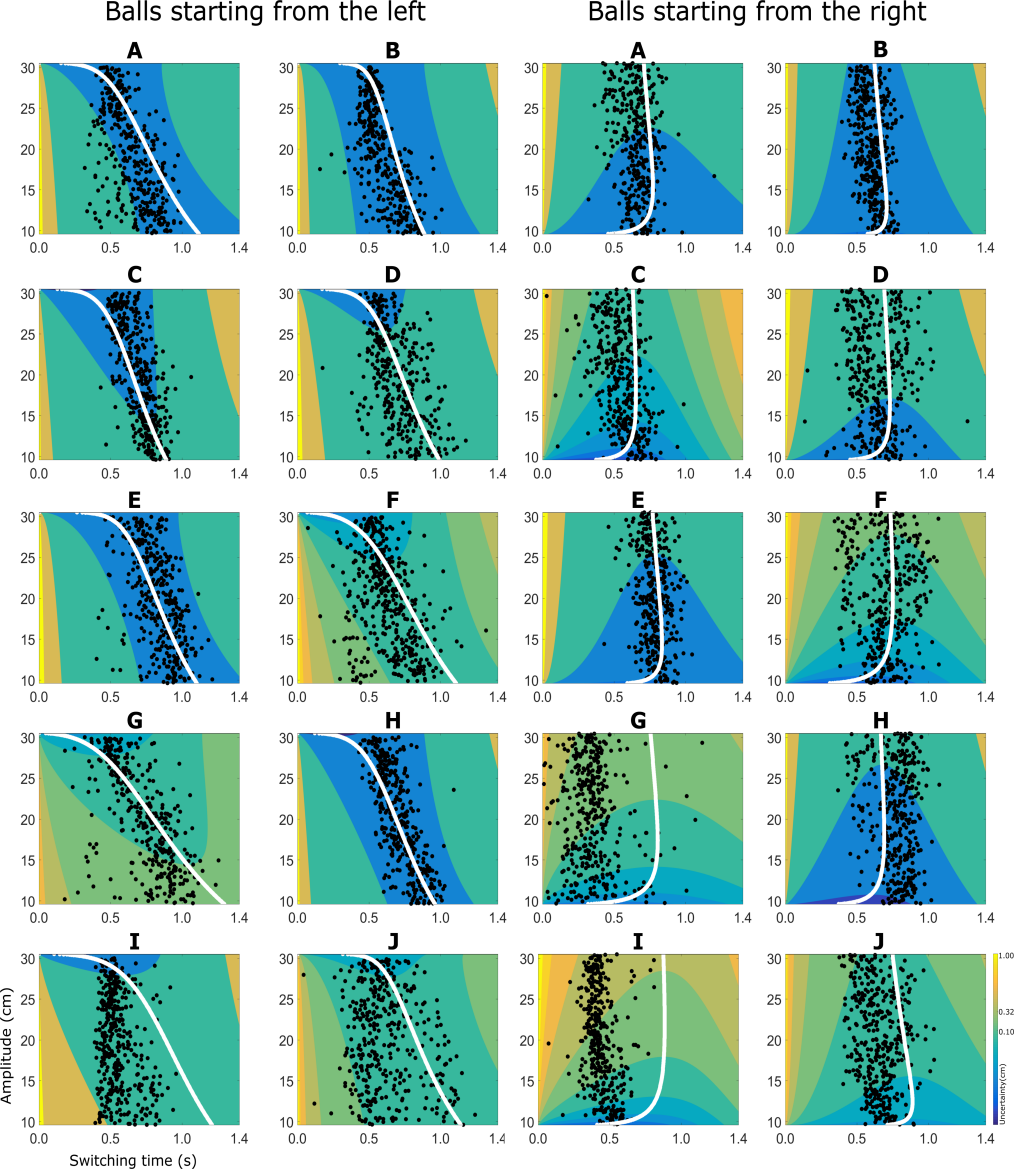
**Sensorimotor uncertainty function and empirical switching times for all ten subjects (A–J), for balls starting from the left and right.** The combined sensorimotor uncertainty is derived from the separately measured sensory and motor uncertainty (see Fig 4) and is here depicted as color coded contours. Data from all subjects is coded using the same color code, warmer colors indicate greater errors. The white curves indicate the valley of the combined uncertainty function and indicates the optimal switching times for the different movement amplitudes. The black dots indicate the actual switching time used by the participants, plotted against the required movement distance for each trial.

Fig 5 shows the combined sensorimotor uncertainty as a function of time and amplitude for the individual subjects, based on the independently derived sensory and motor uncertainties. The predicted optimal switching times and the observed switching times are indicated by the solid white line and the black dots, respectively. Because of the velocity dependence of the sensory uncertainty, the two start positions of the ball result in different sensorimotor uncertainty surfaces and associated optimal switching times. This means that for the same movement amplitude and time, the sensorimotor uncertainty differs for the two start positions. This is because our model of sensory uncertainty factors in the effect of ball velocity, as established by the results of the sensory task.

Even when the required movement amplitude of the paddle is the same, our ideal performer model predicts different optimal switching times for the two conditions: balls starting from the left versus starting from the right (as indicated by the white line). We use a linear regression between the amplitude and switching time to test if the behavioral data shows the same difference as predicted by our ideal performer (see Methods). The linear regressions yielded an average R^2^ of 0.90, (range 0.97–0.72) when the ball starts from the left and an average R^2^ of 0.50 (range 0.84–0.03) when the ball starts from the right.

**Fig 6:**
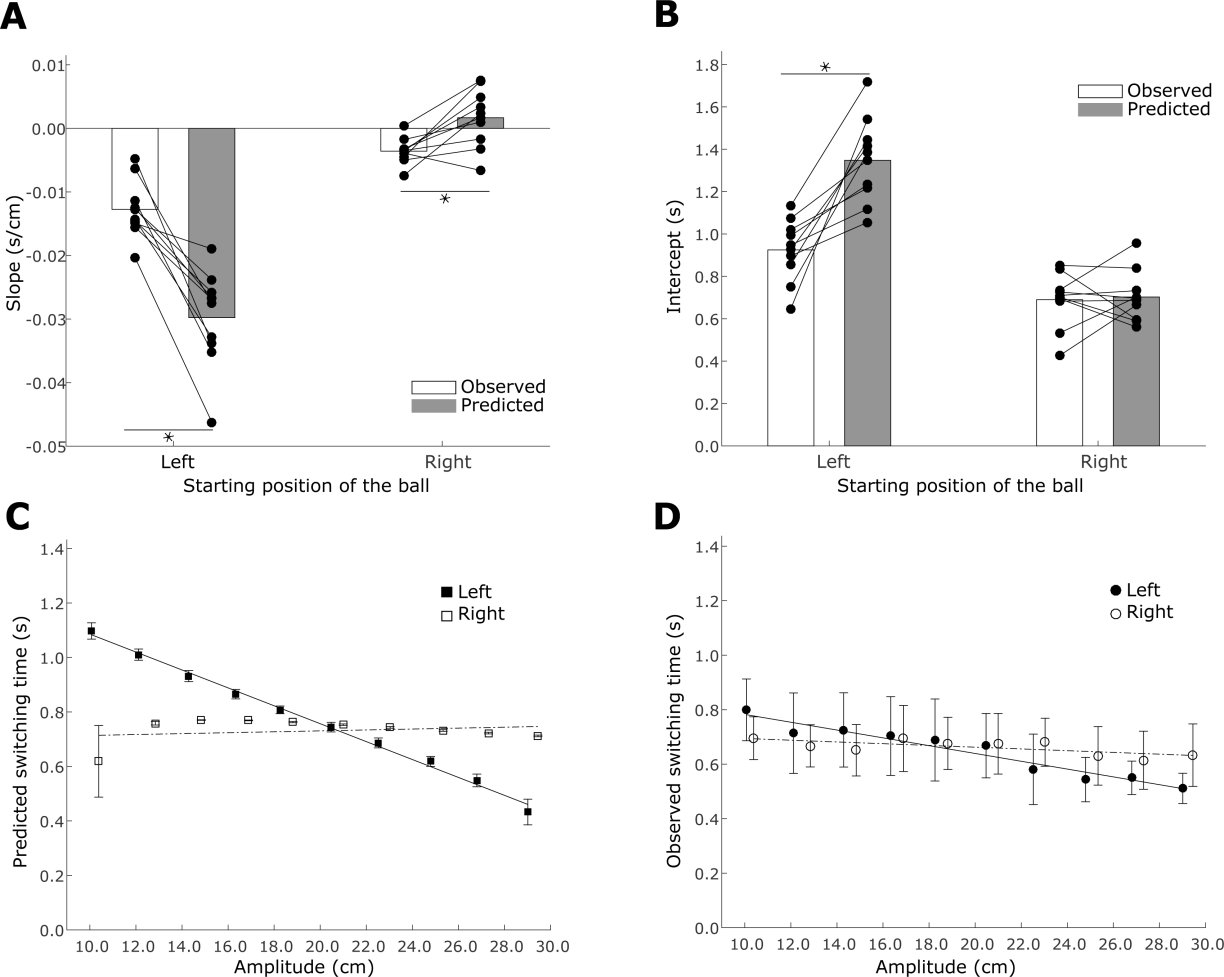
The ideal performer predicts different switching times for the same required movement amplitude but different starting positions of the ball. Participants also choose different switching times between the two conditions. A. The black dots show the regression slopes for the individual subjects, both predicted and observed. B. The black dots represent the predicted and the observed intercepts for the individual subjects. C. The lines represent the linear regression between the amplitude and the predicted switching times for the same representative participant (A). D. Lines show the linear regression between the required movement amplitude and the observed switching times for a representative participant (A). Empirical switching times are shown based on binned data (open and closed dots with standard error), but regression was performed on the raw data.

As shown by the white bars in Fig 6A and 6B, when the ball starts from the left, the linear regression between the switching time and amplitude has a steeper slope (t (18) = -5.86, p<0.005) and larger intercept (t (18) = 3.83, p<0.005) than when the ball starts on the right. This indicates that the observed switching time distributions are different when the ball starts left as compared to the condition when the ball starts from the right. This is further illustrated in Fig 6D, using data from a representative subject (A).

Next, we analyze the predictions of the optimal performer model using the same approach (Fig 6A and 6B gray bars). We generate the distribution of optimal switching times and use a linear regression between the amplitude and predicted switching times. The linear regression yields qualitatively similar results. Fig 6C shows the optimal regression lines for the representative subject (A). Comparing Fig 6C and 6D, we see the qualitative similarity between the slopes of the regression. That is, when the ball starts from the left (solid line), the linear regression between the predicted switching time and amplitude has a steeper slope (t (18) = 11.27, p<0.005) and larger intercept (t (18) = -8.67, p<0.005) than when the ball starts on the right (dashed line).

However, when we compare the predicted behavior with the observed behavior, we find significant differences for all parameters except for the intercept in the condition when the ball starts from the right (ball starting from the left: slope (t(18) = 6.10, p<0.005), intercept (t(18) = -5.40, p <0.005); ball starting from the right: intercept (t(18) = -0.23), p = 0.8, slope (t(18) = -3.34, p< 0.005)). It is important to note that the optimal performer model cannot account for any variability in the switching times for movements with the same amplitude. This is also evident in the length of the error bars in Fig 6C.

### Determinants of switching time variability

A behavioral observation that is not captured by the optimal performer model is the variability in empirical switching time. Pooled across all conditions and subjects, the variability in switching time was 136 ms. However, when we separate the trials based on the ball start position, the average variability in switching time was 152 ms (SE = 10 ms), when the ball started from the left. When the ball started from the right, the average variability in switching times was 120 ms (SE = 9 ms). It is important to highlight that the average movement amplitude and average horizontal velocity of the balls are comparable in both conditions (with different start positions). The only difference is in the sensorimotor uncertainty. This difference in sensorimotor uncertainty can be qualitatively described in terms of the width of the minimal uncertainty area, which is the range of switching times which result in similar sensorimotor uncertainty. This is evident in Fig 5, if we compare the sensorimotor uncertainty for each participant for both the ball starting positions, by comparing the blue (or the minimum uncertainty) region.

Although it is possible that paddle width could affect the variability in switching times, we found no significant effects on switching time variability or errors. Results of an ANOVA using paddle width and direction of the ball as factors do not indicate any effects of the paddle width on the switching time variability (F (3,27) = 0.65, p = 0.6) or the errors (F (3,27) = 0.54, p = 0.7). Similarly, we found no interaction between paddle widths and ball direction on switching time variability (F (3,27) = 0.0013, p = 0.9) or the errors (F (3,27) = 0.47, p = 0.7). In the remainder of the results section, we quantify the patterns observed in the variability of switching time, by evaluating possible determinants as detailed in the methods section.

#### Uncertainty in task outcome

**Fig 7:**
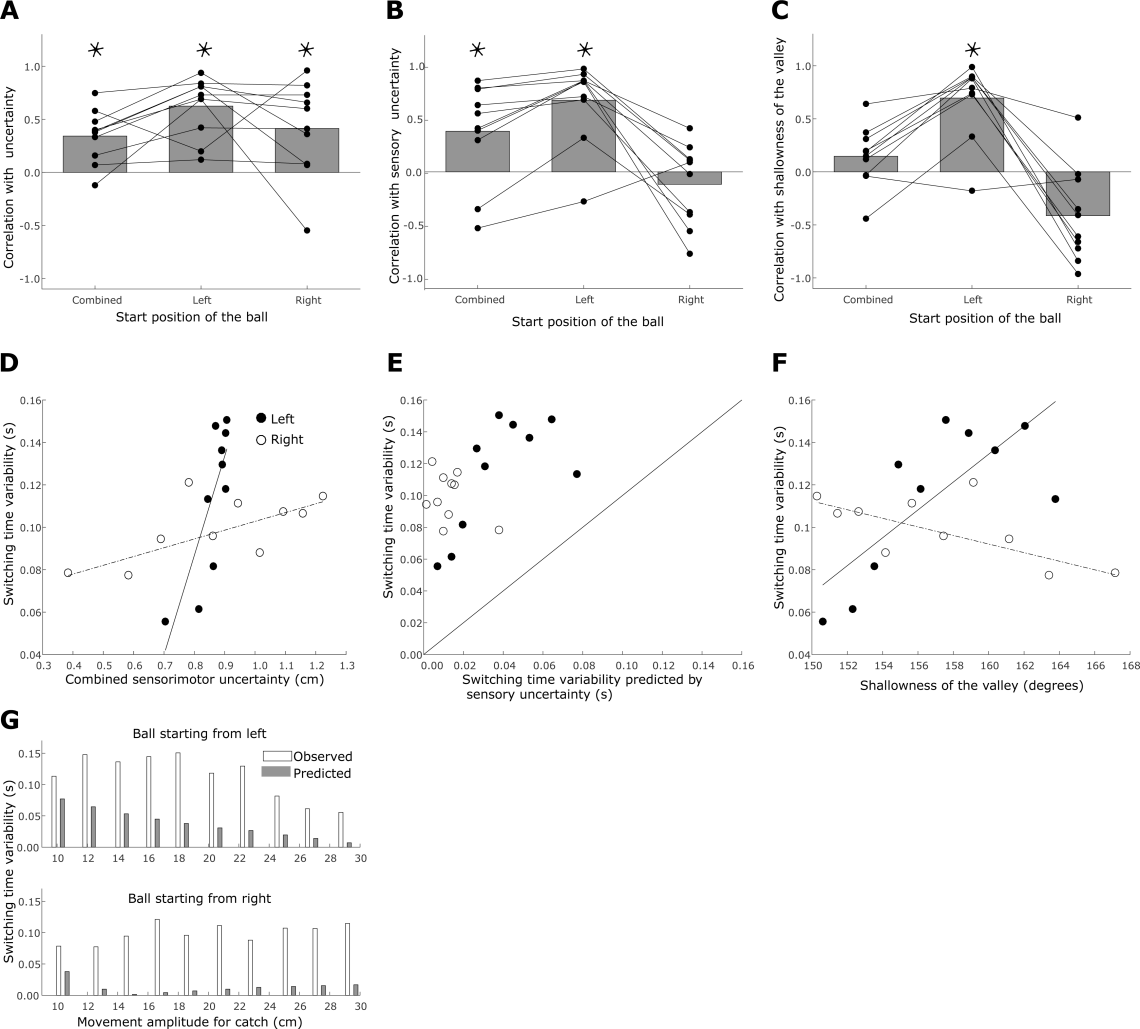
Factors contributing to the variability in the switching time. A. Correlation between the variability in the switching time and the minimum sensorimotor uncertainty (σ_combined_(*t_optimal*)) for both start positions together and separated for the two start positions. Dots corresponding to each participant are joined by a straight line across all the three conditions. B. Correlation between the variability in switching time and the sensory uncertainty (σ_sensory_(*t_optimal*)). C. Correlation between the variability in switching time and the shallowness of the valley. D. Switching time variability versus combined sensorimotor uncertainty for a representative subject (A) for the two start positions of the ball. Each dot results from binned data–based on the horizontal velocity of the ball—to compute switching time variability and average sensorimotor uncertainty. E. Correlation between switching time variability predicted by sensory uncertainty and observed switching time variability for representative subject A. The scatter of the data points above the unity line indicates that the observed variability was always greater than the predicted variability. F. Switching time variability versus shallowness of the valley for representative subject A. G. Switching time variability predicted by sensory uncertainty for representative subject A. The open bars indicate the switching time variability observed in each bin. The shaded bars indicate the variability predicted by the sensory uncertainty about the landing position of the ball, only. Note that increases in movement amplitude are associated with increased horizontal ball velocity for balls starting from the right and decreasing horizontal ball velocities for balls starting from the left.

First, we consider the uncertainty in the task outcome, which is the minimum sensorimotor uncertainty that the subject can attain in each trial ((σ_combined_(*t_optimal*)), see determinant 1 in the methods section). When we analyze both conditions, pooling across the two starting locations of the ball, we find a weak positive correlation between the uncertainty and SD of switching time (as shown in Fig 7A, Pearson correlation coefficient ρ = 0.34 (mean), t(18) = 4.30, p <0.005). We continue to see a positive correlation at the group level when we separate the trials based on the start position of the ball. In the condition when the ball starts from the left we find a positive correlation (Pearson correlation coefficient ρ = 0.62 (mean), t(18) = 7.03, p <0.005). When the ball starts from the right the mean correlation coefficient is 0.41 (Pearson correlation coefficient ρ = 0.41 (mean), t(18) = 2.90, p = 0.009). Qualitatively, the correlation between the uncertainty and the SD of the switching times was stronger in the condition when the ball started from the left (illustrated in Fig 7D with data from a single subject; see Table 2 for individual subject data).

**Table 2:** Correlation of switching time variability with minimum sensorimotor uncertainty.

Participant	Combined	Left	Right
A	0.39	0.81	0.66
B	0.40	0.69	0.60
C	0.07	0.12	0.08
D	0.48	0.94	0.36
E	0.16	0.42	0.41
F	0.75	0.84	0.82
G	-0.12	0.69	-0.55
H	0.58	0.20	0.96
I	0.38	0.81	0.07
J	0.33	0.73	0.73

#### Uncertainty in estimated landing location

Note that the minimum uncertainty contains a sensory uncertainty component, σ_S_(*t_optimal*), which is the uncertainty in the estimation of the landing position. As explained in the methods section, we assume that in a given trial the landing position that subjects use is drawn from a normal distribution. The mean of this distribution is the actual landing position and its standard deviation is given by the sensory uncertainty at the optimal switching time (σ_S_(*t_optimal*)). Therefore, we analyze the explicit contribution of this sensory uncertainty to switching time variability. We compute the range of switching times that may be chosen in each trial based on the sensory uncertainty at the optimal switching time.

Across all subjects only ~10% of the variability in the switching time can be explained by the sensory uncertainty at the optimal switching time (σ_S_(*t_optimal*)). For a representative participant (A), we can compare the variability in switching time that can be explained by the sensory uncertainty alone with the observed variability in switching time by comparing how the dots scatter against the unity line (in Fig 7G we present the same data as bar plots). In principle, if the variability in switching time is a result of only sensory uncertainty, the sensory uncertainty should be able to account for the majority of the variability observed in switching time. Note that, motor uncertainty is measured as the end point uncertainty. Therefore, any contribution of the motor uncertainty is likely to be in the end point, affecting the outcome of the trial as a hit or miss and not in timing of the movement initiation.

Also, greater sensory uncertainty should predict a wider range of switching times and thus the observed variability in switching times should correlate with the predicted range. When we pooled the data of the two conditions, we find that the predicted variability was positively correlated to the observed variability in switching times (Pearson correlation coefficient ρ = 0.38 (mean), t(18) = 2.55, p = 0.02). However, if we segregate the data based on the starting position of the ball, the observed correlations are very different (as shown in Fig 7B). When the ball starts from left, we observed a positive correlation (Pearson correlation coefficient ρ = 0.67 (mean), t(18) = 5.57, p <0.005). By contrast, when the ball starts from the right, at the group level we find no significant correlation between the predicted switching time range and the observed switching time variability. The correlations for individual subjects tend to be negative (see Table 3 for individual subject data). The effect of sensory uncertainty should not be different between the two ball start positions. This suggests that the variability observed in the switching time cannot be satisfactorily explained by the underlying sensory uncertainty.

**Table 3:** Correlation of switching time variability with switching time variability predicted by sensory uncertainty alone.

Participant	Combined	Left	Right
A	0.55	0.68	-0.38
B	0.63	0.71	0.23
C	-0.53	-0.28	0.09
D	0.30	0.86	-0.02
E	0.79	0.86	-0.56
F	0.39	0.85	-0.77
G	0.41	0.86	0.41
H	-0.35	0.32	-0.40
I	0.78	0.92	0.11
J	0.86	0.97	0.12

#### Shallowness of sensorimotor valley

Next, we test the third determinant of variability in the switching time: shallowness of the optimal valley of the sensorimotor function. The shallowness of the optimal valley can be estimated using the angle between the sensory and motor uncertainty curves at the ideal switching time. A larger angle (closer to 180°) is associated with a shallower valley, which allows for more variability in switching time without sacrificing performance. Contrary to what we would expect, if we consider balls starting from left and right together, we find no significant correlation between the shallowness of the valley and variability in switching time (Fig 7C). However, for balls coming from the left, we find, on average, a positive correlation between switching time variability and the shallowness of the valley (Pearson correlation coefficient ρ = 0.70 (mean), t(18) = 6.16, p <0.005). For balls coming from the right, no significant positive correlation is found in any of the subjects (see Table 4 for correlation values for all subjects; data from representative subject (A) in Fig 7F).

**Table 4:** Correlation of switching time variability with shallowness of the valley.

Participant	Combined	Left	Right
A	0.20	0.74	-0.66
B	0.12	0.73	-0.61
C	-0.04	-0.18	-0.07
D	0.31	0.89	-0.35
E	0.15	0.89	-0.41
F	-0.03	0.88	-0.84
G	0.64	0.79	0.51
H	-0.44	0.33	-0.96
I	0.37	0.90	-0.02
J	0.19	0.99	-0.72

#### Regression model to explain variability in switching time

The above analyses show that, in isolation, neither shallowness of the valley nor the minimum uncertainty can satisfactorily explain the variability observed in switching times. Therefore, we use a linear regression, containing both the shallowness of the valley and the minimum uncertainty, to explain the variability observed in switching times. This revealed that eight out of ten subjects have positive coefficients for both minimum uncertainty and shallowness of the valley (average R^2^ = 0.40, range 0.01–0.71. See Table 5 for all the values). Across subjects, there is a positive coefficient for both the minimum uncertainty (t (18) = 3.59, p<0.005) and the shallowness (t (18) = 4.39, p<0.005) of the valley with the variability in switching time.

**Table 5:** Contribution of sensorimotor uncertainty and shallowness of the valley to switching time variability based on regression.

Participant	Constant	Shallowness	Uncertainty	R^2^
	β_0_	β_1_	β_2_	
A	-0.84	0.0052	0.149	0.59
B	-1.53	0.0088	0.278	0.53
C	0.13	-0.0002	0.007	0.01
D	-0.95	0.0062	0.083	0.61
E	-0.61	0.0040	0.092	0.18
F	-0.70	0.0045	0.107	0.71
G	-0.99	0.0069	0.005	0.42
H	0.21	-0.0010	0.062	0.35
I	-0.93	0.0059	0.023	0.38
J	-1.98	0.0115	0.220	0.36

It is important to note that when the ball starts from the right, neither of these factors can sufficiently explain the variability observed in switching time on their own. Unlike the correlation coefficients which cannot disambiguate the role of shallowness of the valley and minimum uncertainty, the regression model results in positive coefficients for both minimum uncertainty and shallowness of the valley. The positive regression coefficients suggest that individually, the shallowness of the valley and the minimum value of the sensorimotor uncertainty correlate with variability in switching time. However, when the ball starts from the right, greater velocity leads to larger amplitude of movement for successful catch. Thus, the sharper valley–owing to steeper sensory and motor uncertainty curves–is also associated with greater minimum uncertainty. Therefore, in a qualitative sense the decrease in variability due to the sharper valley is counterbalanced by the increase in the variability due to greater minimum uncertainty, hence the absence of a correlation.

## Discussion

In this study, we explored three factors–sensory uncertainty and two aspects of the underlying sensorimotor uncertainty function, that might contribute to switching time variability in a sensorimotor trade-off task. We find that the switching time variability is sensitive to the two aspects of the underlying sensorimotor uncertainty function and not to the sensory uncertainty. The sensorimotor uncertainty function has a V-shaped cross-section along the time axis, giving it a valley like appearance. The lowest point of this valley corresponds to the minimum possible uncertainty in the final outcome of the task that an ideal performer can attain–and hence is the optimal switching time. The shallowness of the valley is a measure of the sensitivity of the uncertainty in the outcome of the task to the switching time. Our results suggest that both factors contribute to the observed switching time variability: larger sensorimotor uncertainty is associated with greater switching time variability and the shallowness of the valley is associated with increase in the switching time variability.

Previous studies [2,3] have suggested that the variability in the switching time is a result of a shallow valley, that is, a large change of switching times result in relatively minor changes in sensorimotor uncertainty. Battaglia and Schrater [2]) also suggested an influence of an exploration-exploitation trade-off on switching time variability. Here we quantitatively analyze the contribution of these factors to the switching time variability. This requires a paradigm where minimum uncertainty and shallowness of the valley can be manipulated without the awareness of the subject, such that the behavior is consistent throughout the task. Although such manipulations can be easily achieved in a random dot motion paradigm [2], we choose to use the ball catching paradigm. This choice is motivated by the advantages of the ball catching paradigm put forward by Faisal & Wolpert (2009). The ball catching paradigm allows continuous accumulation of sensory information, as opposed to the discrete appearance of new dots in the random dot task. The ball catching paradigm explores the subjects’ awareness of movement uncertainty over a broad range of movement amplitudes and movement times. Previous studies indicate that the brain has internal models of gravity [19] and uses consistent internal representations of physical laws [20,21], which contributes to the naturalistic appeal of the task. This in turn makes the task more comparable to daily life situations (but see [22] for differences between virtual and real ball catching tasks). Therefore, although the ball catching task employs complex stimuli from the perspective of modeling and interpretation, it is well-suited to elicit natural behavior from the subjects.

Even though the ball catching paradigm has many advantages, the original implementation had one key drawback. Faisal and Wolpert [3] modeled the sensory uncertainty only as a function of time. This choice was possibly driven by the weak effect of velocity that they observed on sensory uncertainty. We wanted to address the effect of velocity and ensure that it does not confound the behavior in the combined task. Therefore, we implemented two key changes in the paradigm. Firstly, we used a larger range of speeds for each session, i.e. 0–15 cm/s as opposed to 0–7.5 cm/s used in their study. Secondly, we moved the start position of the ball to the top corners of the screen and tested subjects for each starting position separately. That is, in one session the ball is projected toward the paddle’s start position and in a second session the ball is projected away from the paddle’s start position. These two modifications allowed us to test for the effect of velocity using a wider range of velocities. Although modeling the sensory uncertainty only as a function of time is valid within the design constraints of the original implementation, it leaves no way of manipulating the sensory uncertainty while keeping the viewing duration constant. That is, if we compare trials which require movements of similar amplitudes, an ideal performer would switch at different switching times depending on how the sensory uncertainty evolves with time. This in turn is a key prediction of the optimal model which can be used to test optimality in the observed behavior. Therefore, by including the effect of ball velocity on sensory variability we resolve an important drawback of the ball catching paradigm—we can manipulate the sensory uncertainty that can be attained for a given viewing duration (by using a range of velocities) and the estimation of the sensory uncertainty is more accurate as compared to Faisal and Wolpert [3].

We predict the ideal switching times by modeling an ideal performer, as described in the previous studies [2,3]. The aim of the ideal performer was to maximize the performance (number of catches) in our experiment. Therefore, the optimal strategy is to minimize sensorimotor uncertainty in each trial, which would maximize the probability of catching the ball. Minimizing the sensorimotor uncertainty as the optimal strategy does not predict any difference for two groups of trials with different minimum uncertainty. That is, irrespective of how high or low the minimum uncertainty is, the optimal strategy is to switch such that combined uncertainty is at its minimum. Naturally, on trials in which the probability of catch is low (i.e. the trials with greater ball velocity and therefore greater minimum uncertainty) minimizing the sensorimotor uncertainty may still not result in catching the ball. Our results indicate that switching time variability is greater in these trials where the probability of catch is low. This is in line with observations in other domains. For example, Pekny, Izawa and Shadmehr [23] reported a reward modulation of the end point variability in reaching movements, suggesting uncertain rewards encourage exploratory behavior. Similarly, studies in reinforcement learning have suggested that uncertainty mediates the exploration-exploitation trade-off to ensure optimal performance [10,11]. Each strategy, exploration or exploitation, is used when it is most likely to be successful. Therefore, the increased variability in switching times in the trials where the minimum uncertainty is high may be a result of the subjects exploring switching times that differ from the predicted optimal switching time so as to improve performance. Battaglia and Schrater [2] first suggested a possible role of exploration-exploitation trade-off in the switching time variability, but their experimental paradigm may not have been ideal to quantify the effects. In this regard, it is important to highlight that the tendency to explore rather than exploit depends on the sensitivity of each individual to the probability of success [10]. That is, how high (or low) the probability of catch (or minimum uncertainty) have to be to elicit exploratory behavior is a very individual trait. This further implies that for subjects with very low sensory uncertainty, exploratory behavior may not be observed at all. This may explain why some subjects do not show significant correlations and why in general the correlation coefficients are low.

It is important to note that in reinforcement learning theory, expected uncertainty is differentiated from unexpected uncertainty [24,25]. In our task, it may be argued that the subject is aware of the uncertainty in the task outcome, and that therefore this uncertainty is expected and should not result in exploratory behavior. However, the combined task is such that the subjects initiate movement towards an uncertain estimate of the landing position of the ball. How this uncertainty in the target affects the planning of the movement to catch the ball is not clear. Exploring switching times near the optimal switching time may be an attempt at exploring different kinematics for the same intended endpoint of the reaching movement. Also, it is possible that the subjects have uncertainty about their own uncertainty estimates.

Our results indicate that variability in the switching time decreases as the sensitivity of the task outcome to switching time variability increases. Both Battaglia and Schrater [2] and Faisal and Wolpert [3] have suggested that the sensitivity of the task outcome affects variability in switching time. In this study, we quantify the sensitivity of the sensorimotor uncertainty to the variability in switching time using shallowness of the valley as a measure. Given that the aim of the ideal performer is to maximize performance, a flat minimum or a shallow valley implies that precise choice of optimal switching time would result in relatively small improvement in performance as compared to the case when the subjects are broadly near the predicted optimum. On similar lines, [26]) describe how in complex real-life tasks, the computational intractability of the optimal solution may result in approximations. Such approximations may even be more useful while acting under time constraint (like in [27]) because the cost of time devoted to accumulation of information is not constant across the trial [28]. This suggests that to maximize performance, an approximate or “near” optimal switching time suffices and computing the exact optimal switching time is unnecessary.

In our data, shallowness of the valley and minimum uncertainty explain variability in switching time. The effect of shallowness of the valley on the variability in switching time implies a sensitivity to error (distance between the paddle and the ball at the end of each trial). On the other hand, the effect of minimum uncertainty suggests a role of reward (indicated by binary score in our experiment). Recent studies have explored the role of both error-based feedback and reward-based feedback on variability of movement. Cashaback, McGregor, Mohatarem and Gribble [29]) reported that subjects rely on error-based feedback more than reinforcement-based feedback while planning reaching movements. Earlier Izawa and Shadmehr [30] have shown that reinforcement-based learning becomes increasingly more important when the quality of error feedback deteriorates. In line with these results, variability in switching always increases as the minimum uncertainty increases. When the ball starts from the left trials with greater minimum uncertainty have shallower valleys, hence both factors add to the variability. In contrast, when the ball starts from the right, both shallowness and minimum uncertainty cannot explain the variability in switching time on their own. This is because when the ball starts from the right, trials with greater minimum uncertainty have sharper valleys. Qualitatively, the increase in variability due to the greater uncertainty is nullified by the decrease in variability due to the sharper valleys. Therefore, to understand the individual effect of the minimum uncertainty and shallowness of the valley, we use the regression model. The regression model, essentially, pools the data of both the conditions together. The positive regression coefficients suggest a positive influence of both the shallowness of the valley and the minimum uncertainty to switching time variability. In other words, it suggests that switching time variability is both driven by error and by reward signals.

The study of sensorimotor trade-off, by means of segregating the sensing stage from the acting stage, is a novel approach to understand how the sensory and motor systems interact to generate a given choice. The segregation of the sensing and acting stages artificially enforces a serial processing, although in many natural situations perception and action partially overlap or evolve parallel to each other, as evidenced by behavioral as well as neurophysiological studies [31–33][34]. The enforced serial processing helped, here and in previous studies [2,3], to establish that subjects represent sensory and motor variability in order to optimize task performance. How this is implemented in the nervous system and whether or not this involves a unified representation of sensorimotor uncertainty, as raised by Battaglia and Schrater [2], remains an open question. Potentially, the evaluation of sensorimotor trade-off in pathological conditions, as advocated by Faisal and Wolpert [3], may elucidate this and related questions.

In conclusion, we have provided additional evidence that subjects optimally trade-off sensory and motor uncertainty using a time-constrained ball catching task. Furthermore, the variability in switching from sensing to acting is best captured by the shape of the sensorimotor uncertainty surface. The variability in switching time increases if performance is less sensitive to changes in switching time. Also, the variability in switching time increases as the total uncertainty about a successful task outcome. Although, we could not provide conclusive quantitative evidence for an exploratory process, the patterns observed in variability in switching times are consistent with an exploratory process. In this perspective, Parkinson’s disease represents an interesting testing ground, both because of dopaminergic influences on the temporal coupling of deciding and acting [35,36] and because of such influences on the exploration-exploitation trade-off.
